# New thermoplastic poly(carbonate-urethane)s based on chain extenders with sulfur atoms

**DOI:** 10.1007/s11696-016-0112-5

**Published:** 2016-12-22

**Authors:** Magdalena Rogulska, Anna Kultys, Andrzej Puszka

**Affiliations:** Department of Polymer Chemistry, Faculty of Chemistry, Maria Curie-Skłodowska University, ul. Gliniana 33, 20-614 Lublin, Poland

**Keywords:** Sulfur-containing thermoplastic polyurethanes, Aliphatic–aromatic chain extenders, Thermal properties, Mechanical properties, Adhesive and optical properties

## Abstract

New thermoplastic segmented polyurethanes were obtained by a one-step melt polyaddition using 40, 50 and 60 mol% poly(hexane-1,6-diyl carbonate) diol of $\bar{M}_{n} = 860$ g mol^−1^, 1,1′-methanediylbis(4-isocyanatobenzene) and 2,2′-[sulfanediylbis(benzene-1,4-diyloxy)]diethanol, 2,2′-[oxybis(benzene-1,4-diylsulfanediyl)]diethanol or 2,2′-[sulfanediylbis(benzene-1,4-diylsulfanediyl)]diethanol as a chain extender. FTIR, atomic force microscopy, differential scanning calorimetry and thermogravimetry were used to examine the polyurethanes’ structure and thermal properties. Moreover, their Shore *A*/*D* hardness, tensile, adhesive and optical attributes were determined. They were transparent high-molar-mass materials showing good tensile strength (up to 51.9 MPa). The polyurethanes exhibited improved adhesion to copper taking into consideration that of conventional ones, and middle or high refractive index values (1.57–1.60), and both these parameters increased with an increase of the content of sulfur atoms in the polyurethane chain. The newly obtained polyurethanes can be considered as materials for numerous medical and optical appliances.

## Introduction

Polyurethanes, owing the variety of their uses, have an important place in the world production of plastics. They can be either thermoplastic or thermosetting. Thermoplastic polyurethanes are usually produced by the reaction of diisocyanates with both oligomeric and short-chain diols, resulting in block copolymers of the (A–B)_n_ type. One block of the polymer chain, called the soft segment, is constituted by oligomeric diol, while the second block, referred to the hard segment, is built of a diisocyanate and short-chain diol. The soft segments confer the polyurethanes with their softness, elasticity, long elongation at break and low-temperature resistance, whereas the hard segments particularly affect the modulus of elasticity, hardness and tear strength. Oligoester, oligoether or oligocarbonate diols are the most used oligomeric diols to synthesize conventional thermoplastic polyurethanes. The polyurethanes based on the latter diols, which are relatively new, have at the same time a high resistance to heat, hydrolysis and oxidation in comparison with polyurethanes derived from oligoether and oligoester diols (Sonnenschein 2014; Resiak and Rokicki 2000; Kojio et al. 2009; Špirková et al. 2012; Hrdlička et al. 2014).

The best mechanical properties of these polyurethanes were observed when the hard segments were formed by 1,1′-methanediylbis(4-isocyanatobenzene) (MDI) and butane-1,4-diol (BD) (Wirpsza 1993). In special cases, e.g., to obtain polyurethanes with higher modulus of elasticity and hardness, enhanced thermal stability or liquid-crystalline properties, BD is replaced by nonconventional chain extenders, among others, 2,2′-(benzene-1,4-diyldioxy)diethanol, bisphenol-S, 6,6′-oxybis(2-aminobenzothiazole), imide-based diols, mesogenic diols, including derivatives of biphenyl, benzophenone and azobenzene (Wirpsza 1993; Liaw 1997; Yeganeh et al. 2009; Mehdipour-Ataei and Mahmoodi 2014; Padmavathy and Srinivasan 2003). The object of our study is thermoplastic polyurethanes containing sulfur atoms. They are introduced to a polyurethane chain using diols with sulfide linkages and dithiols (Rogulska et al. 2007, 2013, 2015; Kultys et al. 2008, 2009, 2012; Kultys and Rogulska 2011; Kultys and Puszka 2013, 2014; Rogulska and Kultys 2016). These polyurethanes can show improved adhesive properties to metals (Kultys and Puszka 2013, 2014; Rogulska et al. 2013, 2015; Rogulska and Kultys 2016), increased refractive index (Kultys and Puszka 2013, 2014; Rogulska and Kultys 2016) and enhanced antimicrobial activity (Kultys and Puszka 2014) in relation to those of conventional ones based on aliphatic chain extender.

This paper deals with the synthesis and characterization of new thermoplastic segmented poly(carbonate-urethane)s (PCURs) based on aliphatic–aromatic diols containing one, two or three sulfur atoms, i.e., 2,2′-[sulfanediylbis(benzene-1,4-diyloxy)]diethanol (OSOE), 2,2′-[oxybis(benzene-1,4-diylsulfanediyl)]diethanol (SOSE) or 2,2′-[sulfanediylbis(benzene-1,4-diylsulfanediyl)]diethanol (SSSE), 1,1′-methanediylbis(4-isocyanatobenzene) (MDI) and poly(hexane-1,6-diyl carbonate) diol (PHCD) of $\bar{M}_{n} = 860$ g mol^−1^. Using the above-mentioned reactants, we expected to achieve transparent PCURs with good mechanical strength, showing at the same time specific properties resulting from the presence of sulfur atoms. Such materials can be attractive for numerous medical applications like blood tubing, catheters, cannulae, pacemakers, neurostimulators and orthopedic nail encapsulation (Resiak and Rokicki 2000; Choi et al. 2011), as well as for optical applications like ophthalmic lenses, fiber optics and non-linear optics, and so on (Jha et al. 2008). They can also be used as interlayers or inner layers for automobile laminated windscreens (Hepburn 1992) and printed wiring boards (Hirano et al. 2007).

## Experimental

### Materials

The OSOE diol (m.p. = 101–102 °C after recrystallization first from methanol/water and next from 1,2-dichloroethane) was prepared from 4,4′-sulfanediyldiphenol and ethylene carbonate by a modified previous method (Penczek et al. 1993). Diols SOSE (m.p. = 95–96 °C after recrystallization from benzene) and SSSE (m.p. = 75–76 °C after recrystallization from benzene) were obtained by the reaction of suitable dithiols with 2-bromoethanol in ethanolic solution of sodium hydroxide according to the procedure given in the literature (Wdowicka and Podkoscielny 1996). PHCD of $\bar{M}_{n} = 860$ g mol^−1^ was acquired from Sigma-Aldrich (USA). Prior to being used, the PHCD was heated at 90 °C in vacuo for 10 h, while MDI (98%) from Sigma-Aldrich (Germany) was used as received.

### Synthesis

**Scheme 1:**
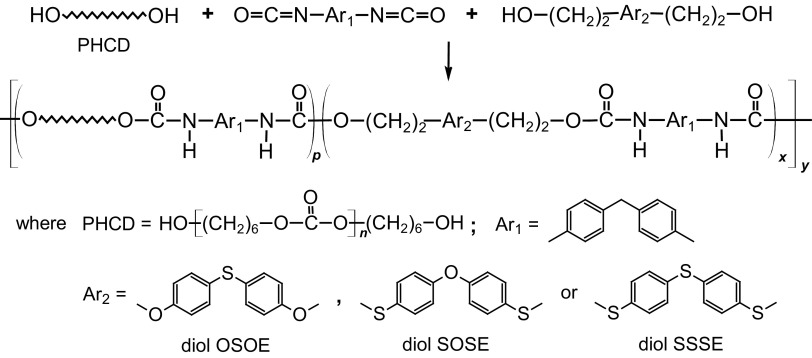
Synthesis of PCURs

The PCURs, containing 40, 50 or 60 mol% of the soft segment, were synthesized via a one-step melt polyaddition from MDI, OSOE, SOSE or SSSE and PHCD (Scheme 1) using an equimolar ratio of the dihydroxyl compounds to the diisocyanate according to the following procedure.

At the beginning, the diols OSOE, SOSE or SSSE and PHCD (0.01 mol together) were placed in a three-necked round-bottom flask fitted with a mechanical stirrer, a gas inlet tube and a calcium chloride drying tube, and then heated to 110 °C in an oil bath while being stirred under dry nitrogen until clear melt appeared. Next, MDI (0.01 mol) was added and the reaction was continued until the viscosity increase made stirring impossible. Then, the reaction temperature was slowly increased to 135 °C and in general the hard product obtained was kept at this temperature for 2 h.

### Measurement methods

The measurements of reduced viscosities (*η*_red_s, dL g^−1^) were conducted with an Ubbelohde viscometer (Poland) at 25 °C. The PCUR samples were dissolved in a phenol (Ph)/1,1,2,2-tetrachloroethane (TChE) (Ph/TChE) mixture with a mass ratio of 1:3 at a concentration of 0.5 mass%.

The Fourier transform infrared (FTIR) spectra were recorded with a Bruker Tensor 27 FTIR spectrometer (Germany) using the attenuated total reflectance (ATR) technique. The samples were thin films. All spectra were obtained at room temperature after averaging 32 scans between 600 and 4000 cm^−1^ with a resolution of 4 cm^−1^ in the absorbance mode.

The ultraviolet–visible (UV–VIS) spectra were collected with a UV-1800 (Shimadzu, Japan) UV spectrophotometer in the range of 300–900 nm, with a sampling interval of 0.5 nm. The PCURs were in the form of the compression-molded 1-mm-thick sheets.

Thermogravimetry (TG) was performed with a Netzsch STA 449 F1 Jupiter thermal analyzer (Germany) under the following operational conditions: the heating rate of 10 °C min^−1^, a dynamic atmosphere of helium (flow 20 cm^3^ min^−1^), temperature range of 40–1000 °C, sample mass ~10 mg, sensor thermocouple type S TG-DSC. All TG measurements were taken in Al_2_O_3_ crucibles. As a reference, an empty Al_2_O_3_ crucible was used.

Differential scanning calorimetry (DSC) was carried out using a Netzsch DSC 204 calorimeter (Germany) operating in a dynamic mode. The dynamic scans were performed at the heating/cooling rate of 10 °C min^−1^ under argon atmosphere (flow 30 cm^3^ min^−1^) from −100 to 200 or 220 °C. The mass of the sample was ~10 mg. As a reference, an empty aluminum crucible was applied. The reported transitions came from the first and second heating scans. Glass-transition temperatures (*T*_g_s) of the samples were defined as the inflection point on the curves of the heat-capacity changes, and the melting temperatures (*T*_m_s) as endothermic peak maxima.

Atomic force microscopy (AFM)-modulus imaging was utilized to determine the local elastic modulus of the sample surfaces. Images were obtained using a MultiMode™8 AFM microscope with a NanoScope^®^ V (Bruker-VEECO, USA) Controller using PeakForce Quantitative Nanomechanical Mapping mode. The local elastic modulus was determined using the Derjaguin–Muller–Toporov (DMT) modulus model. RTESPA (Bruker) probe that was used (silicon tip on nitride lever) had a nominal spring constant of 40 N m^−1^. The cuttings from crude polymers after 1-month conditioning at room temperature were used as specimens.

The Shore hardness tests were carried out with a Zwick 7206/H04 durometer (Germany), type A and D. The readings were taken after 15 s at the temperature of 23 °C.

Tensile properties were measured on five replicates of each material using a Zwick/Roell Z010 (Germany) machine in accordance with Polish Standard PN-81/C-89034 (EN ISO Standard 527-1:1996 and 527-2:1996) at the speed of heads of 100 mm min^−1^ at 23 °C. One mm thick and 6 mm wide tensile test samples (for the section measured), cut from the pressed sheet, were applied.

A Carver hydraulic press (USA) was used for press molding of the polymers at 130–175 °C under a pressure of 10–30 MPa.

The measurements of the single lap shear strength of the polymers to a copper plate sized 100 mm × 25 mm × 1.5 mm were taken using a Zwick/Roell Z010 tensile-testing machine (Germany) following Polish Standard PN-EN 1465:2009. The adhesive joint, 12.5 mm × 25 mm × 0.2 mm, was prepared by pressing the polymer between the ends of two copper plates at 155–175 °C (in accordance with PN-EN-13887:2005), and then leaving them under a pressure of 30 MPa to cool to room temperature. Next, the plates were fixed by tensile-testing machine clips and underwent tensile testing, the speed of heads of 2 mm min^−1^ at 23 °C.

Refractive index measurements were done at 23 °C by a Conbest Abbe’s Refractometer Type 325 instrument (Poland) in keeping with method A of European Standard EN ISO 489:1999. 1-Bromonaphtalene was used between the sample film and the prism shield.

## Results and discussion

**Fig. 1:**
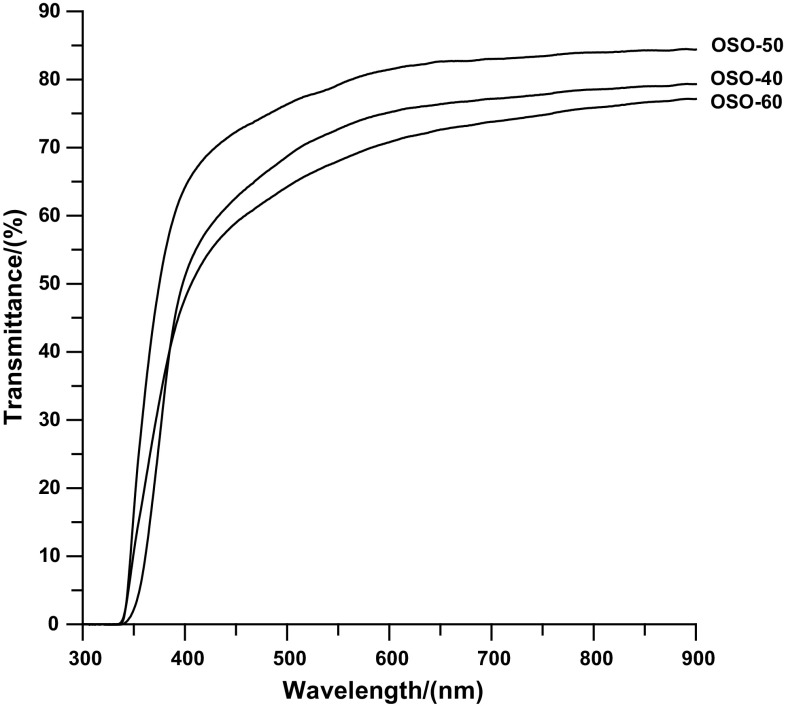
UV–VIS spectra of the PCURs based on diol OSOE

The PCURs were colorless or light-yellow materials, generally showing good transparency. Transmittances at 800 and 500 nm determined for these polyurethanes were contained in the range of 67.1–84.0% and 43.7–76.3%, respectively (see Table 1 and Fig. 1).

**Table 1:** Designations, *η*_red_, transmittance and refractive index data of the PCURs

PCUR	Diol	Soft-segment content/mol%	Hard-segment content/mass%	*η*_red_/dL g^−1^	Transmittance/%		Refractive index
					*T*_500_^a^	*T*_800_^a^	
OSO-40	OSOE	40	55.8	–^b^	68.8 ± 0.51	78.5 ± 0.36	1.573 ± 0.0008
OSO-50		50	48.4	2.60 ± 0.052	76.3 ± 0.85	84.0 ± 0.91	
OSO-60		60	41.9	1.91 ± 0.033	64.2 ± 0.72	75.9 ± 0.61	
SOS-40	SOSE	40	56.3	1.23 ± 0.049	61.6 ± 0.52	78.1 ± 0.40	1.582 ± 0.0007
SOS-50		50	48.9	2.78 ± 0.038	74.5 ± 0.74	81.8 ± 0.63	
SOS-60		60	42.4	2.17 ± 0.029	56.0 ± 0.95	72.8 ± 0.65	
SSS-40	SSSE	40	57.1	0.96 ± 0.021	43.7 ± 0.67	67.1 ± 0.42	1.602 ± 0.0008
SSS-50		50	49.6	1.12 ± 0.043	47.8 ± 0.44	72.5 ± 0.38	
SSS-60		60	43.1	1.98 ± 0.025	65.9 ± 0.56	83.6 ± 0.48	

^a^Transmittance data at 500 and 800 nm, respectively

^b^PCUR insoluble in a Ph/TChE mixture and other solvents

The obtained PCURs revealed different resistance levels against common organic solvents. The least resistant were those based on SSSE diol; they dissolved at room or elevated temperature in *N*-methyl-2-pyrrolidone, *N*,*N*-dimethylacetamide, *N,N*-dimethylformamide, TChE and a Ph/TChE mixture. The other samples, with the exception of OSO-40, were completely soluble at room or elevated temperature in TChE, and at room temperature in a Ph/TChE mixture. The *η*_red_ values found for these PCURs, contained in the range of 0.96–2.78 dL g^−1^, suggest their high-molar masses.

Table 1 shows designations, *η*_red_, transmittance and refractive index data of the PCURs.

**Fig. 2:**
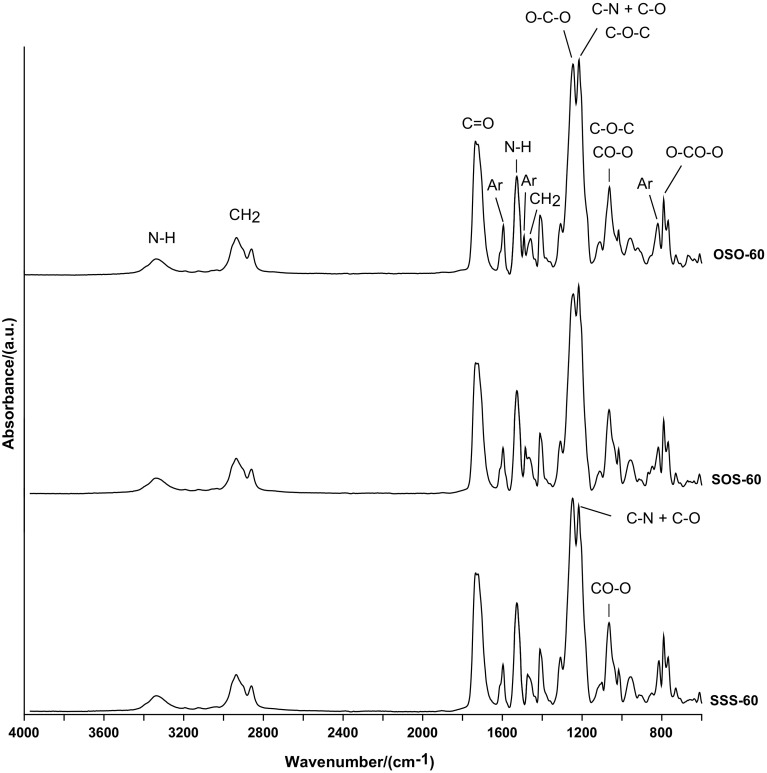
ATR-FTIR spectra of the PCURs with 60 mol% soft-segment content

The chemical structures of all the PCURs were endorsed by ATR–FTIR spectroscopy. The main absorption bands can be found below, whereas representative spectra are given in Fig. 2.

ATR-FTIR (cm^−1^): 3339–3318 (N–H stretching) and 1529–1526 (N–H bending) of the urethane group; 1736–1734 (non H-bonded C=O stretching) of the urethane and carbonate groups; 1721–1720 (H-bonded C=O stretching) of the urethane and carbonate groups; 1254–1241 (O–C–O stretching of the carbonate group); 1218–1214 (coupled C–N and C–O stretching of the urethane group and for PCURs from diols OSOE and SOSE also asymmetric C–O–C stretching in aliphatic–aromatic or aromatic ether); 1063–1062 (symmetric CO–O stretching of the urethane and carbonate groups and for PCURs from diol OSOE also symmetric C–O–C stretching in aliphatic–aromatic ether); 791–790 (out-of-plane bending) of O–CO–O; 2937–2933 and 2860–2858 (asymmetric and symmetric C–H stretching) and 1485–1457 (asymmetric C–H bending) of CH_2_; 1597–1594 (C–C stretching) of benzene ring; 820–813 (C–H bending) of *p*-disubstituted benzene ring.

### DSC

Table 2 gives the numerical data [*T*_g_, *T*_m_ and heat of melting (Δ*H*) values] obtained for all the PCURs and, for comparison’s sake, also for PHCD soft segment and regular polyurethanes (RPURs) OSO, SOS and SSS examined earlier (Rogulska et al. 2015), which were derived from MDI and suitable diols, and are the models of hard segments in these PCURs. From the presented data, it follows that the PCURs showed relatively high *T*_g_s (10–46 °C), which points to the high phase mixing of hard and soft segments, with the highest values exhibited by polyurethanes of OSO series. Generally, in each series they lowered as PHCD content increased.

**Table 2:** DSC data of the polymers

Polymer	*T*_g_/ °C		*T*_m_/ °C		Δ*H/*J g^−1^	
	I^a^	II^a^	I^a^	II^a^	I^a^	II^a^
OSO^b^		89	105; 159, 168		1.0; 32.1	
OSO-40	41	46				
OSO-50	30	32	178		1.3	
OSO-60	21	22	160, 187		2.5	
SOS^b^		64	104; 129; 195	189	0.7; 3.2; 40.3	37.7
SOS-40	10	35	178, 191		24.4	
SOS-50	23	25	117		9.4	
SOS-60	16	15				
SSS^b^		63	107; 138, 169, 184	182	0.6; 41.1	35.3
SSS-40	34	36	155		8.1	
SSS-50	24	26	128		3.7	
SSS-60	18	16	121		1.0	
PHCD	–69	–63	10, 30	31	55.5	37.2

^a^I and II, first and second heating scans, respectively

^b^OSO, RPUR from diol OSOE; SOS, RPUR from diol SOSE; SSS, RPUR from diol SSSE

**Fig. 3:**
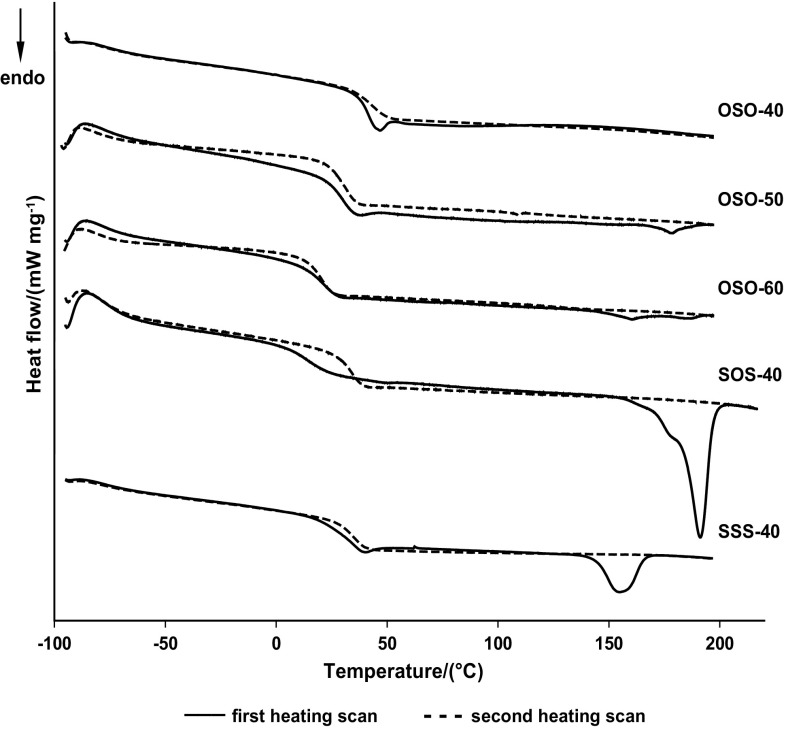
DSC curves of PCURs OSO-40, OSO-50, OSO-60, SOS-40 and SSS-40

DSC curves of PCURs OSO-40 and SOS-60 from the first heating scans did not reveal endothermic peaks. On this basis, the amorphous structure of these polymers can be predicted. The curves of the other PCURs, however, exhibited endothermic peaks in the range of 117–187 °C, connected with the melting of the hard-segment domains. Taking into account Δ*H* values of these transitions, one should conclude that these PCURs, with the exception of SOS-40, were characterized by a low degree of ordering. A low tendency of all these PCURs to form ordered structures was confirmed by the lack of endothermic peaks on DSC curves during the second heating scans. Figure 3 presents the curves received for selected PCURs.

Amorphous structures and relatively high *T*_g_s (16–30 °C) were also found for previously obtained similar PCURs from different chain extender with sulfur atoms, i.e., [methanediylbis(benzene-1,4-diylmethanediylsulfanediyl)]diethanol (Kultys et al. 2012). On the other hand, significantly lower *T*_g_s (–40–9 °C) and generally higher degree of ordering were received in the case of the analogous polyurethanes with oligoether soft segment (Rogulska et al. 2015).

### AFM

**Fig. 4:**
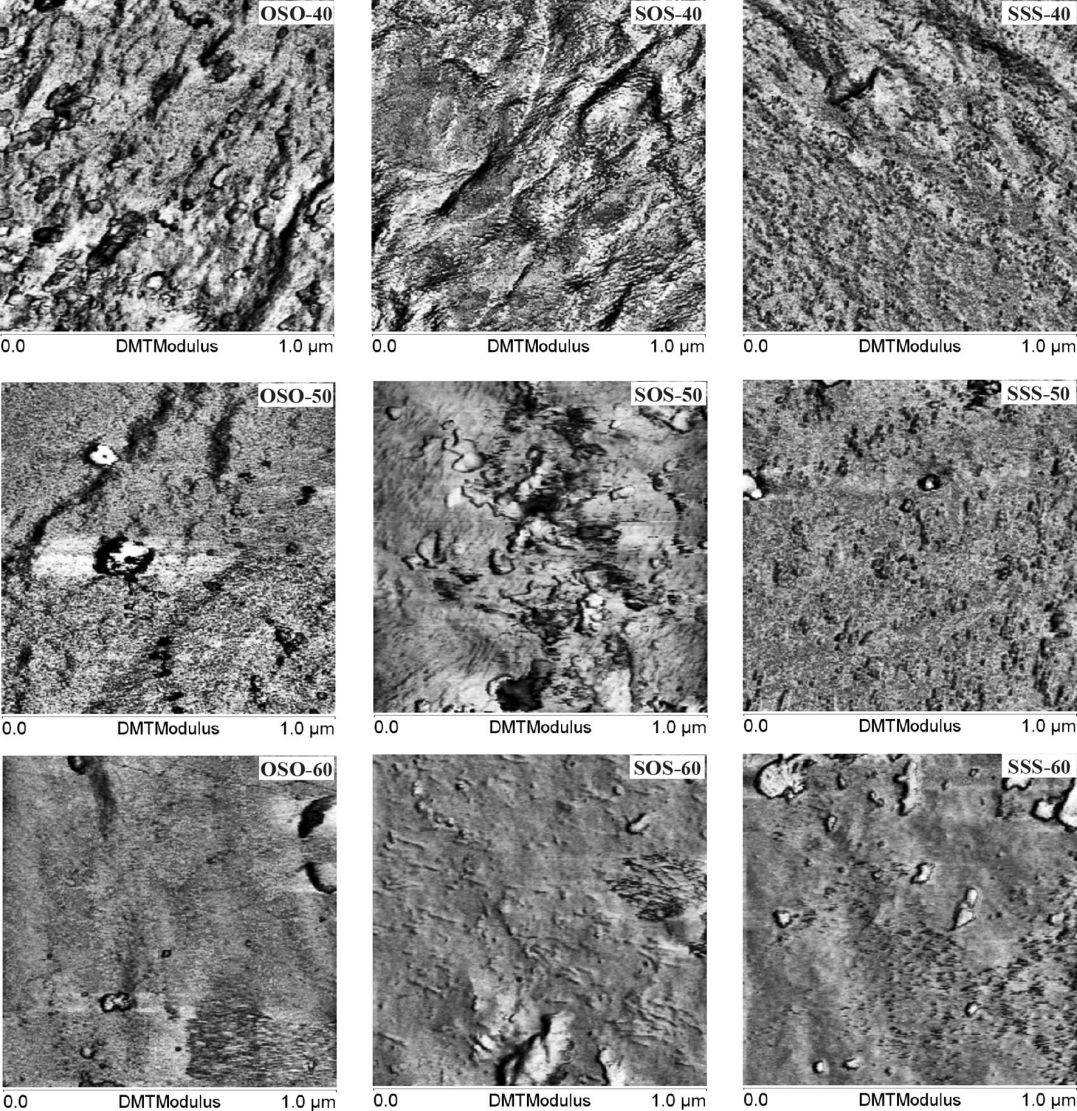
DMT modulus AFM images of the PCURs

A heterogeneous bulk morphology of the investigated PCURs was verified by the DMT modulus AFM images, shown in Fig. 4. The dark areas of the lower modulus stand for the soft-segment-rich domains whereas the bright ones of the higher modulus correspond to the hard-segment-rich domains. In each series, images with the highest contrast can be seen for the PCURs with the highest content of the hard segments. In the case of sample SOS-40 bright, parallel oriented rod-like structures are visible, contrary to the other samples where shorter, rather randomly oriented features are observed. It points to the highest degree of ordering of this polymer within hard-segment-rich domains. It is in agreement with DSC data. The process of decrease in hard-segment content resulted in the diminishing of the contrast of the images obtained. It can be caused by the formation of shorter sequences of hard segments, making aggregation difficult, and in consequence the creation of phases with smaller difference in the modules.

### TG

Table 3 presents the TG data obtained for the PCURs as well as the RPURs and PHCD. All the PCURs were characterized by relatively good and similar thermal stability, even though, as was expected, it was worse than the stability of the analogous polyurethanes containing oligoether soft segment (Rogulska et al. 2015). Considering the *T*_1_ values, they were stable up to 280–286 °C. Slight differences also appeared in the values of the remaining temperature mass loss indicators (*T*_5_, *T*_10_ and *T*_50_). However, it may be noticed that the SOS and SSS series PCURs exhibited somewhat higher *T*_1_s and these values were much higher (above 20 °C) than those obtained for the respective hard-segment-type RPURs. This indicates that in the case of PCURs of those series the mutual stabilization effect of hard and soft segments occurred (Chattopadhyay and Webster 2009).

**Table 3:** TG data of the polymers

Polymer	*T*_1_^a^/ °C	*T*_5_^b^/ °C	*T*_10_^c^/ °C	*T*_50_^d^/ °C	*T*_max_^e^/ °C
OSO^f^	280	310	324	365	370, 405
OSO-40	280	308	320	359	347, 391, 430
OSO-50	282	309	322	358	358, 390, 430
OSO-60	283	308	320	354	354, 396, 430
SOS^f^	261	303	316	344	341, 405
SOS-40	284	306	316	356	355, 388
SOS-50	284	306	317	351	355, 388
SOS-60	286	309	322	353	355, 385
SSS^f^	258	306	319	349	345, 405
SSS-40	284	307	319	353	352, 376
SSS-50	282	307	319	352	354, 386
SSS-60	286	310	322	354	355, 381
PHCD	209	264	290	352	364

^a,b,c,d^The temperature of 1, 5, 10 and 50% mass loss from the TG curve, respectively

^e^The temperature of the maximum rate of mass loss from the differential TG (DTG) curve

^f^OSO, RPUR from diol OSOE; SOS, RPUR from diol SOSE; SSS, RPUR from diol SSSE

**Fig. 5:**
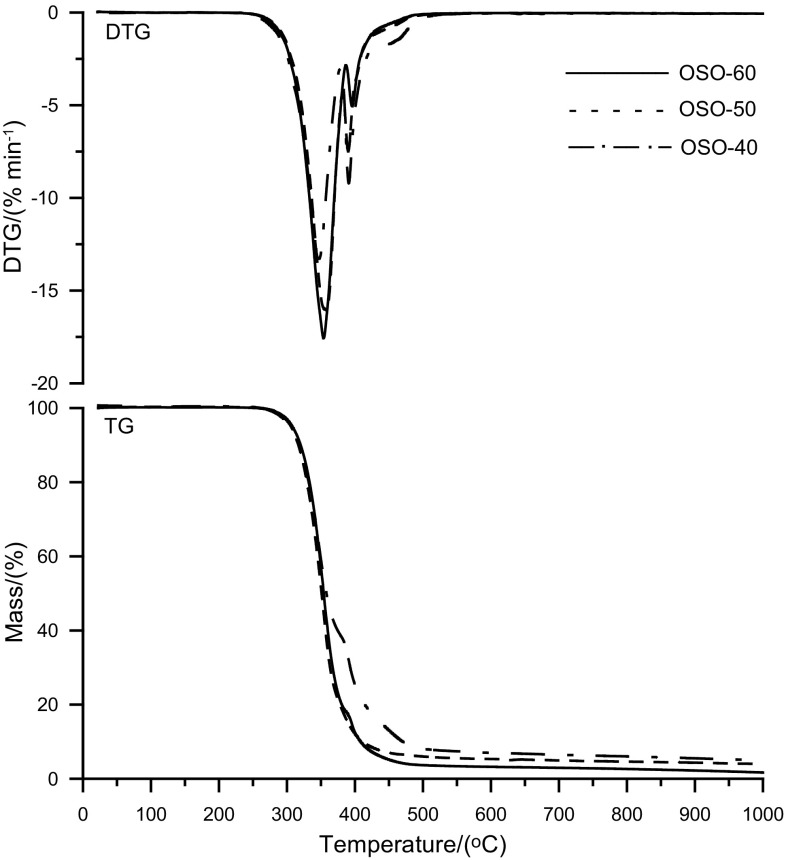
DTG and TG curves of the PCURs based on diol OSOE

The OSO series PCURs were decomposed in three stages. The DTG curves (see Fig. 5) showed two relatively sharp peaks with maxima at 347–358 °C and 390–396 °C, and a small intense and broad peak visible as a shoulder with the accepted maximum at 430 °C. The first peak showing the highest intensity may be ascribed to the decomposition of the urethane and carbonate linkages. The second peak, as well as the shoulder, whose intensity decreased with the increase of soft-segment content, should be related to the further decomposition of the hard segments. The decomposition of ether and diaryl sulfide linkages, as well as aromatic compounds probably occurred in these stages (Rogulska et al. 2015).

The PCURs of the remaining two series were decomposed in two stages. The DTG curves showed, similarly as in the case of OSO series, two sharp peaks with maxima at 352–355 °C and 376–388 °C, but they did not exhibit the peak observed as a shoulder. The second peak, of much lesser intensity, also decreased with the increase of soft-segment content, except that it appeared at lower temperature. Less stable aryl–alkyl sulfide linkages in the structure of these polyurethanes may be responsible for this feature.

### Mechanical properties

**Fig. 6:**
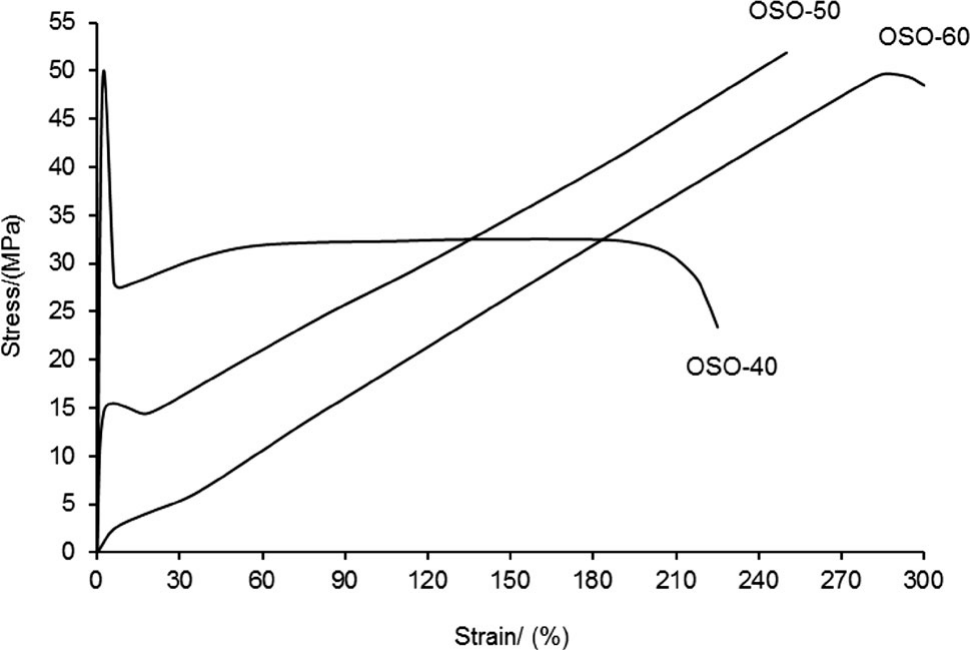
Stress–strain curves of the PCURs based on diol OSOE

As is evident from the data given in Table 4, PCURs with the lowest soft-segment content (OSO-40, SOS-40 and SSS-40) as well as OSO-50 showed high values of the modulus of elasticity (192–880 MPa) and hardness (95–97 °Sh A, 49–64 °Sh D). Their stress–strain curves showed yield stress, and in the case of OSO-40 it refers to maximal stress (tensile strength). Both values of the modulus of elasticity and the course of the stress–strain curves allow to classify these polymers as plastics. The other samples were characterized by a much lower modulus of elasticity (3.00–43.4 MPa), typical of polyurethane elastomers and lower hardness (75–87 °Sh A, 25–37 °Sh D). Tensile strength of all the PCURs was in the range of 28.5–51.9 MPa and decreased in the following series order OSO > SSS > SOS, while their elongation at break was similar and contained in the range of 200–325%. Increased soft-segment content in each series resulted in decreased hardness and the modulus of elasticity, whereas elongation at break increased. As an example, Fig. 6 presents the stress–strain curves of all the PCURs based on diol OSOE.

**Table 4:** Mechanical properties of the PCURs

PCUR	Hardness/°Sh		Modulus of elasticity/MPa	Tensile strength/MPa	Elongation at break/%	Pressing temperature/ °C
	*A*	*D*				
OSO-40	96 ± 0.7	64 ± 0.5	880 ± 26	50.0 ± 1.8	225 ± 11	155
OSO-50	97 ± 0.5	49 ± 0.4	192 ± 19	51.9 ± 1.3	250 ± 13	145
OSO-60	79 ± 0.3	31 ± 0.6	7.23 ± 0.28	49.7 ± 1.0	300 ± 22	130
SOS-40	95 ± 0.5	60 ± 1.0	460 ± 11	34.4 ± 0.69	225 ± 14	175
SOS-50	84 ± 0.5	37 ± 0.3	27.6 ± 1.8	36.6 ± 0.89	275 ± 15	165
SOS-60	77 ± 0.4	26 ± 0.7	3.00 ± 0.18	28.5 ± 0.55	325 ± 10	165
SSS-40	97 ± 0.3	58 ± 0.3	650 ± 33	40.0 ± 0.77	200 ± 12	165
SSS-50	87 ± 0.3	33 ± 0.6	43.4 ± 2.1	39.9 ± 1.1	250 ± 20	160
SSS-60	75 ± 0.4	25 ± 0.5	4.00 ± 0.25	38.4 ± 0.92	300 ± 28	150

The newly obtained polyurethanes containing oligocarbonate soft segments possessed higher tensile strengths (28.5–51.9 vs. 15.6–43.9 MPa) and lower elongations at break (200–325 vs. 320–710%) than the analogous ones containing oligoether soft segments described earlier (Rogulska et al. 2015). Specially great increase in tensile strength was recorded for polyurethanes with 60 mol% soft-segment content. But if one compares the PCURs described here with the familiar PCURs based on [methanediylbis(benzene-1,4-diylmethanediylsulfanediyl)]diethanol (Kultys et al. 2012), it should be concluded that the enhanced tensile strengths occurred only in the case of OSO series with similar elongations at break.

### Adhesive properties and refractive index

**Fig. 7:**
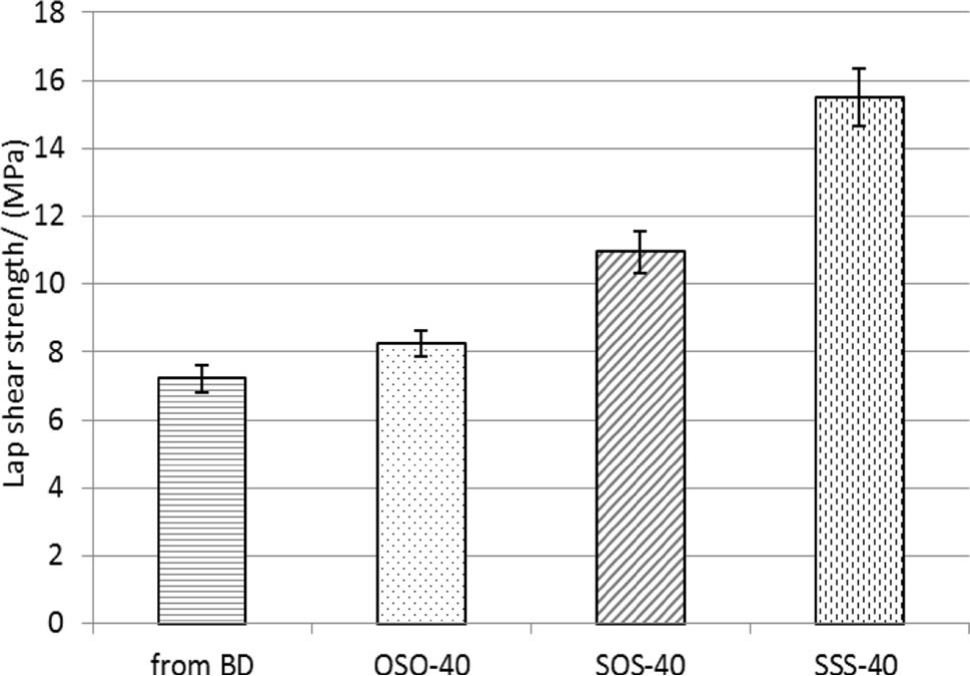
Lap-shear strength of the PCURs with 40 mol% soft-segment content and their analog obtained from BD

The influence of the kind of sulfur-containing chain extender on the lap shear strengths to copper (adhesion) and refractive index of the obtained PCURs was examined for samples with 40 mol % soft-segment content (the highest sulfur content), i.e., OSO-40, SOS-40 and SSS-40. As shown in Fig. 7, adhesion was parallel to the increase in the number of sulfur atoms in the chain extender used (the shear strengths from 8.3 to 15.5 MPa) and was better than that obtained for the analogous PCUR based on BD as a chain extender (7.2 MPa). The replacement of the previously used oligoether soft segment (Rogulska et al. 2015) to oligocarbonate one also had beneficial influence on the shear strengths.

Refractive index (see Table 1), similarly as adhesion, increased with the increase in the number of sulfur atoms in the chain extender used. Owing to the fact that the polyurethane derived from BD was non-transparent, a suitable comparison for this parameter was impossible. In view of the classification that applies to the plastic lens industry, the determined refractive index values should be considered as middle or high (Jang et al. 2009). It is worth noting that these values are higher than those received for similar sulfur-containing polyurethanes (Kultys and Puszka 2013; Rogulska and Kultys 2016).

## Conclusions

The obtained PCURs were transparent high-molar-mass materials showing amorphous structures or with a small degree of ordering within hard-segment domains. They exhibited a low degree of microphase separation; their *T*_g_s were in the range of 10–46 °C and generally lowered as the soft-segment content increased. On the basis of TG data, we discovered that the thermal stability of the PCURs was relatively good and slightly depended on the kind of the chain extender used and the soft-segment content. A higher influence of the kind of the chain extender was revealed in the case of mechanical properties. The OSO series PCURs possessed very good tensile strength (49.7–51.9 MPa), somewhat worse of SSS series (38.4–40.0 MPa), and the worst of SOS (28.5–36.6 MPa). The rise of soft-segment content caused a decrease of hardness, the modulus of elasticity and an increase of elongation at break. The PCURs were characterized by middle or high refractive index values (1.573–1.602) and improved adhesion to copper in relation to those of conventional ones (8.3–15.5 vs. 7.2 MPa), and both these parameters increased with an increase of the content of sulfur atoms in the polyurethane chain.

These polyurethanes combine good mechanical strength and a relatively good thermal stability with middle or high refractive index values and good adhesion to copper. It should be stressed that in this aspect they are better than the polyurethanes previously presented by us based on sulfur-containing diol-chain extenders. In this way, the potential application possibilities of this polymer group become wider.
